# “BioProzessTrainer” as training tool for design of experiments

**DOI:** 10.1186/1753-6561-5-S8-P62

**Published:** 2011-11-22

**Authors:** Ralf Pörtner, Oscar Platas-Barradas, Janosh Gradkowski, Richa Gautam, Florian Kuhnen, Volker C Hass

**Affiliations:** 1 Institute of Bioprocess and Biosystems Engineering, Hamburg University of Technology Hamburg, D-21073, Germany; 2 Institute of Environmental and Bio-Technology, Hochschule Bremen, D-28119, Germany

## Concept

Design and optimization of cell culture processes requires intensive studies based on “Design of experiments”-strategies. In academia teaching of DoE-concepts is often insufficient, as in most cases only simple culture strategies (*batch*) can be performed, as time and money are limited. More complex tasks such as feeding strategies for *fed batch* culture can be discussed theoretically only.

To close this gap the virtual “BioProzessTrainer”, a model based simulation tool, was developed. It supports biotechnological education with respect to process strategies, bioreactor control, kinetic analysis of experimental data and modeling. Along with a set of examples for different control and process strategies (*batch*, *fed batch*, chemostat etc.) learners are prepared for real experiments [1,2].

**Figure 1:**
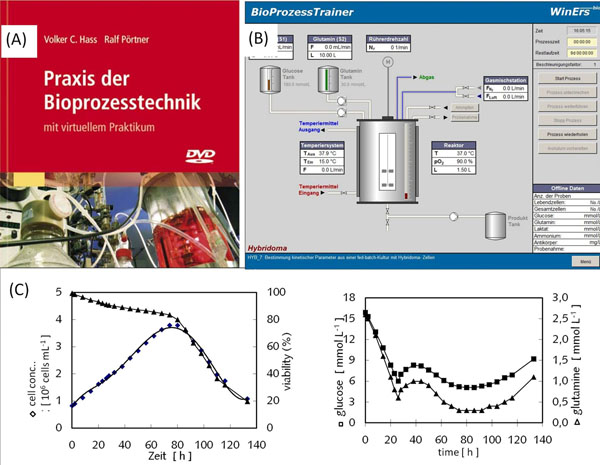
(A) Teaching material: theoretical back-ground, exercises, sample solution [1] (B) Screen of „BioProzessTrainer“ (C) Example: *fed-batch* process with fixed feed rate perfomed with the BioProzessTrainer

The “BioProzessTrainer” (Figure 1) helps to improve the quality of education by using interactive learning forms and by transmitting additional knowledge and skills. Costs for practical experiments can be minimized by reducing plant operation costs. Here a concept for teaching DoE-concepts for batch- (optimization of e.g. substrate concentrations and inoculation cell density) and fed-batch-processes (evaluation and optimization of feeding strategy) using the “BioProzessTrainer” is shown.

## Example 1

*DoE for impact of glucose and glutamine concentration during batch* (*1*,*5 L*) *on cell density and antibody concentration of a mammalian cell line*

Experimental design:

➣ Seed concentration: 4E8 cells/L [±10%]

➣ Glucose conc.: low 15 mmol/L; high 30 mmol/L

➣ Glutamine conc.: low 1 mmol/L; high 4 mmol/L

➣ Culture time: 24h

To induce an experimental error, the seed concentration was varied by +- 10 %. Results see Table 1

**Table 1:** DoE performed with the BioProzessTrainer

			cell conc. [ 10^8^ cells/L]		antibody conc. [mg/L]	
seed conc. [10^8^ cells/L ]		glutamine [mmol/L]	glucose [mmol/L]			
			15	30	15	30
set	4.0	1	7.71	8.01	11.8	12.6
-10%	3.6	1	7.15	7.49	11.1	12.1
+10%	4.4	1	8.23	8.49	12.3	13.0
set	4.0	4	1.17	1.37	22.5	27.7
-10%	3.6	4	1.06	1.24	20.6	25.2
+10%	4.4	4	1.27	1.49	24.4	30.2

Impact of glucose and glutamine concentration on cell density and antibody concentration

Analysis via statistical tools:

➣ One-dimensional ANOVA with respect to glucose at high glutamine concentrations: glucose conc. not significant for cell conc. (p=0.1), significant for antibody conc. (p=0.044); level of significance 0.05

➣ Two-dimensional ANOVA with repetition: interaction between glucose and glutamine conc. not significant for cell conc. (p=0.14); significant for antibody conc. (p=0.046); level of significance 0.05

## Example 2

*DoE for impact of feed rate for glucose and glutamine feed during fed batch* (*constant feed rate*) *on cell density and antibody concentration of a mammalian cell line*

Experimental design:

➣ Seed concentration: 8E8 cells/L

➣ Glucose conc. in glucose feed: 180 mmol/L

➣ Glutamine conc. in glutamine feed: 30 mmol/L

➣ Start feed: 24h; start volume 1.5 L; final volume 3 L

➣ Feed rate glucose / glutamine feed: low 0.02 mL/min; high 0.08 mL/min

Results see Table 2

**Table 2:** Impact of feed rate for glucose and glutamine feed during fed-batch (constant feed rate) on cell density and antibody concentration

	cell conc. [10^9^ cells/L]		antibody conc. [mg/L]	
glutamine feed rate [mL/min]	glucose feed rate [mL/min]			
	0.02	0.08	0.02	0.08
0.02	2.10	2.15	84.2	63.0
0.08	2.95	3.10	67.0	133

Analysis via statistical tools:

➣ Two-dimensional ANOVA without repetition: glucose feed rate not significant for cell conc. (p=0.295) and antibody conc. (p=0.699); glutamine feed rate significant for cell conc. (p=0.035) and not for antibody conc. (p=0.653); level of significance 0.05
